# Immune Profiling To Predict Outcome of Clostridioides difficile Infection

**DOI:** 10.1128/mBio.00905-20

**Published:** 2020-05-26

**Authors:** Mayuresh M. Abhyankar, Jennie Z. Ma, Kenneth W. Scully, Andrew J. Nafziger, Alyse L. Frisbee, Mahmoud M. Saleh, Gregory R. Madden, Ann R. Hays, Mendy Poulter, William A. Petri

**Affiliations:** a Division of Infectious Diseases and International Health, Department of Medicine, University of Virginia Health System, Charlottesville, Virginia, USA; b Division of Biostatistics, Department of Public Health Sciences, University of Virginia Health System, Charlottesville, Virginia, USA; c Department of Public Health Sciences, University of Virginia Health System, Charlottesville, Virginia, USA; d Division of Gastroenterology and Hepatology, Department of Medicine, University of Virginia Health System, Charlottesville, Virginia, USA; e Department of Pathology, University of Virginia Health System, Charlottesville, Virginia, USA; Rutgers University

**Keywords:** *Clostridium difficile*, *Clostridioides*, inflammation, mortality, predictive modeling

## Abstract

There is a pressing need for biomarker-based models to predict mortality from and recurrence of Clostridioides difficile infection (CDI). Risk stratification would enable targeted interventions such as fecal microbiota transplant, antitoxin antibodies, and colectomy for those at highest risk. Because severity of CDI is associated with the immune response, we immune profiled patients at the time of diagnosis. The levels of 17 cytokines in plasma were measured in 341 CDI inpatients. The primary outcome of interest was 90-day mortality. Increased tumor necrosis factor alpha (TNF-α), interleukin 6 (IL-6), C-C motif chemokine ligand 5 (CCL-5), suppression of tumorigenicity 2 receptor (sST-2), IL-8, and IL-15 predicted mortality by univariate analysis. After adjusting for demographics and clinical characteristics, the mortality risk (as indicated by the hazard ratio [HR]) was higher for patients in the top 25th percentile for TNF-α (HR = 8.35, *P* = 0.005) and IL-8 (HR = 4.45, *P* = 0.01) and lower for CCL-5 (HR = 0.18, *P* ≤ 0.008). A logistic regression risk prediction model was developed and had an area under the receiver operating characteristic curve (AUC) of 0.91 for 90-day mortality and 0.77 for 90-day recurrence. While limited by being single site and retrospective, our work resulted in a model with a substantially greater predictive ability than white blood cell count. In conclusion, immune profiling demonstrated differences between patients in their response to CDI, offering the promise for precision medicine individualized treatment.

## Abstract (precis)

Clostridioides difficile infection is the most common health care-associated infection in the United States with more than 20% patients experiencing symptomatic recurrence. The complex nature of host-bacterium interactions makes it difficult to predict the course of the disease based solely on clinical parameters. In the present study, we built a robust prediction model using representative plasma biomarkers and clinical parameters for 90-day all-cause mortality. Risk prediction based on immune biomarkers and clinical variables may contribute to treatment selection for patients as well as provide insight into the role of immune system in C. difficile pathogenesis.

## INTRODUCTION

Clostridioides difficile infection (CDI) has emerged as a leading cause of nosocomial diarrhea and an important public health threat. CDI causes an estimated half million infections and at least 13,000 deaths annually (1, 2). Health care costs associated with CDI management have been estimated to be around $40 billion per year in the United States (3, 4). Thus, the development of novel approaches to treat and prevent CDI is essential.

Although a major risk factor for CDI is antibiotic-associated dysbiosis, other factors, including the use of gastric acid-suppressing agents, nonsteroidal anti-inflammatory drugs, chemotherapy, inflammatory bowel disease, and prolonged hospital stay, are shown to play a role (5). Clinical manifestations associated with CDI range from asymptomatic colonization and mild diarrhea to toxic megacolon and life-threatening fulminant colitis (6). Toxins A and B are major virulence factors of C. difficile that disrupt the cytoskeletal structure and tight junctions of target cells, leading to cell rounding and death. The emergence of a hypervirulent strain, BI/NAP1/027, has altered traditional epidemiology. This strain is capable of producing a binary toxin (C. difficile toxin [CDT]) in addition to toxins A and B and has been implicated in C. difficile outbreaks associated with increased morbidity and mortality since the early 2000s (7–10).

Recent mouse and human studies have shown that C. difficile toxins as well as pathogen-associated molecular patterns (PAMPs) elicit a multifaceted immune response that can impact the disease outcome (11–14). For example, early recruitment of neutrophils and eosinophils (15–17), gamma interferon (IFN-γ)-producing type 1 innate lymphoid cells (18), leptin (19), and microbiota-dependent interleukin 33 (IL-33) (20) were associated with protection. On the other hand, IL-23 (21, 22), IL-17A (23, 24), Toll-like receptor 2-mediated signaling (16), chemokine (C-X-C motif) ligand 5 (CXCL-5) (25), IL-6 (23), IL-8 and C-C motif chemokine ligand 5 (CCL5) (26) were associated with unfavorable outcome during CDI.

A picture is therefore emerging that the immune response to C. difficile is a predominant factor determining clinical outcome. While a limited number of studies have evaluated systemic biomarkers in humans with CDI (24, 26–28), this is the first study to apply multiple immune biomarkers in a model to predict mortality. Since CDI is a result of an exaggerated host immune response, we hypothesized that systemic cytokine signature during CDI can be modeled to predict survival as well as recurrence.

## RESULTS

### Population characteristics.

Plasma samples from a total of 341 CDI patients were included in this study. Baseline patient characteristics are described in Table 1. The median age was 63 years, 50.7% patients were female, and the majority of the patients were of European descent. Severe CDI was present in 34% patients based on a peak white blood cell (WBC) count of >15,000/μl (29) within 48 h of CDI diagnosis. Some patients (13%) had received immunosuppressive therapy at some point within a 90-day period preceding CDI diagnosis.

**TABLE 1:** Demographics and clinical characteristics of CDI inpatients at the University of Virginia hospital^*a*^

Demographic or clinical characteristic	Value for patients		
	All	Moderate CDI	Severe CDI
No. of patients	341	218	123
% Patients		65.8	34.2
Females (%)	50.7	54.5	46.8
Median age (yr) (25th to 75th percentile)	63 (51.2−72)	63 (51−72)	65 (52−73)
Race (%)			
Whites	77.8	76	79.5
Blacks	21	23	18
Others	<1	<1	<1
Mean BMI (SD)	28 (±7.7)	28 (±8)	27.7 (±7.4)
Median Charlson score (25th to 75th percentile)	3 (1−7)	3 (1−7)	3 (1−7)
Mean WBCC (SD)	13.6 (±8.3)	8.7 (±3.6)	22.9 (±6.8)
90-day all-cause death (%)	13	9.6	17.2
30-day all-cause death (%)	8	3.7	14.7
% of ICU patients	30	22.3	42.3
% receiving immunosuppressive therapy*	12.8	12.8	13

a Abbreviations: CDI, Clostridioides difficile infection; BMI, body mass index; WBCC, white blood cell count; SD, standard deviation; ICU, intensive care unit; *, medical record searched from 90 days prior to 30 days post detection.

### Association between plasma cytokine levels and CDI severity.

A total of 341 plasma samples were analyzed (actual sample size numbers for each cytokine varied due to missing values). As shown in Table 2, plasma levels of seven analytes were higher in the severe CDI group (WBC count > 15,000/μl). These analytes included five proinflammatory cytokines: MIF (macrophage migration inhibitory factor) (*P* < 0.0001), IL-6 (*P* < 0.0001), IL-1β (*P* = 0.004), IL-16 (*P* = 0.01) and IL-15 (*P* = 0.04). HGF (hepatocyte growth factor) (*P* < 0.0001) and type 2 cytokine IL-4 (*P* = 0.03) were also significantly elevated. Ninety-day mortality was associated with elevated IL-6, IL-15, sST-2 (suppression of tumorigenicity 2 receptor), IL-8 and TNF-α (tumor necrosis factor alpha) and decreased CCL-5, CCL-4 and EGF (epidermal growth factor) (see Table S1 in the supplemental material).

**TABLE 2:** Plasma cytokine levels in moderate versus severe CDI patients^*a*^

Biomarker	Moderate CDI (WBCC ≤ 15 × 10^9^/liter)	Severe CDI (WBCC > 15 × 10^9^/liter)	*P* value
HGF	252.6 (3,155.7−513.9)	584.1 (286.8−1,166)	<0.0001
MIF	12,055 (5,363−25,898)	23,935 (9,642−42,371)	<0.0001
IL-6	7.73 (2.72−22.67)	16.60 (5.94−35.96)	<0.0001
IL-1β	1.43 (1.07−2.78)	2.46 (1.07−5.58)	0.004
IL-16	674.5 (394.8−1,177)	941.1 (516.5−1,315)	0.01
IL-4	16.5 (14.6−62.7)	29.21 (14.6−58.05)	0.03
IL-15	2.21 (1.41−3.39)	2.6 (1.67−3.85)	0.04
EGF	171.7 (94.5−272)	190.3 (110.3−329)	0.06
sST-2	169,458 (41,260−488,414)	207,281 (71,536−636,327)	0.057
IL-23	4.88 (4.88−14.7)	4.88 (4.88−21.78)	0.20
CCL-4	2,223 (1,686−2,698)	2,036 (1,729−2,677)	0.54
IL-8	69.36 (44.21−117.5)	82.66 (47.22−140.8)	0.23
TNF-α	7.36 (4.61−11.84)	7.28 (4.71−13.7)	0.81
IL-17A	0.32 (0.12−0.79)	0.35 (0.08−1.17)	0.87
IL-10	4.8 (3−5.4)	4.68 (2.99−7.67)	0.56
Eotaxin	529.2 (324.3−819.5)	500.5 (326.8−720.3)	0.30
CCL-5	35,807 (23,050−50,219)	33,487 (18,726−55,342)	0.68

a Patients were stratified into moderate or severe (WBC count of >15,000 per ml) CDI groups based on the Infectious Diseases Society of America (IDSA) recommendations. Plasma biomarker levels are expressed as medians (25th to 75th percentiles). Values for undetectable samples were set to the lowest standard value for the respective target. Statistical significance was calculated by the Mann-Whitney test. The sample numbers (*n*) for biomarkers were as follows: *n* = 345 for HGF, MIF, IL-4, EGF, IL-16, IL-10 and eotaxin; *n* = 326 for IL-6, IL-15 and TNF-α; *n* = 333 for IL-1β; *n* = 326 for sST-2; *n* = 302 for CCL-4, IL-8 and CCL-5; *n* = 283 for IL-17A and IL-23.

10.1128/mBio.00905-20.2 TABLE S1 Descriptive statistics of biomarkers by mortality at 90 days. Plasma cytokine levels (in picograms per milliliter) were compared and are shown as medians (25th to 75th percentiles). Values for undetectable samples were set to the lowest standard value for the respective target. Statistical significance was calculated using Mann-Whitney test. Download Table S1, PDF file, 0.2 MB. Copyright © 2020 Abhyankar et al. 2020 Abhyankar et al. This content is distributed under the terms of the Creative Commons Attribution 4.0 International license.

### Predictive biomarkers of survival.

**FIG 1:**
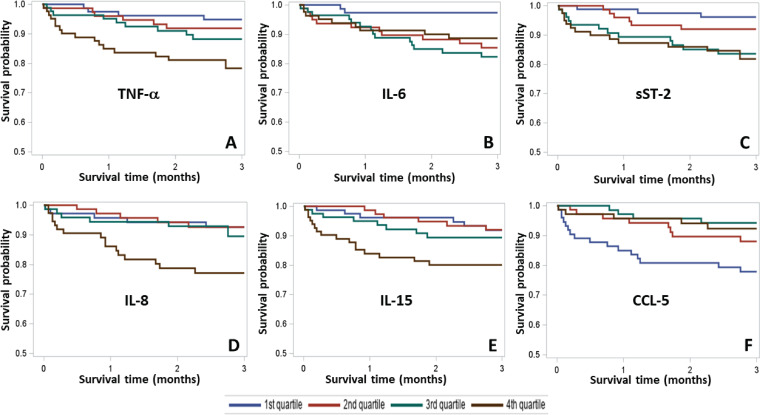
Kaplan-Meier survival curves for biomarker quartiles. Patients were divided into lower quartile (blue), second quartile (red), third quartile (green), and top quartile (brown) for comparison based on the levels of biomarkers in plasma. The relationship of biomarker quartiles with 90-day survival time is shown. (A) TNF-α, (B) IL-6, (C) sST-2, (D) IL-8, (E) IL-15, and (F) CCL-5. The *y* axes represent survival probability. Abbreviations: TNF-α, tumor necrosis factor alpha; IL-6, interleukin 6; CCL-5, C-C motif chemokine ligand 5; sST-2, suppression of tumorigenicity 2 receptor.

Higher levels of TNF-α (fourth quartile, *P* = 0.006), IL-6 (second, third, and fourth quartiles, *P* = 0.03), sST-2 (third and fourth quartiles, *P* = 0.01), IL-8 (fourth quartile, *P* = 0.009) and IL-15 (fourth quartile, *P* = 0.03) were indicative of an increased risk for mortality (Fig. 1A to E). In contrast, higher levels of chemokine CCL-5 (second, third, and fourth quartiles, *P* = 0.008) were associated with better survival (Fig. 1F). Multivariable Cox regression analysis indicated clinical parameters, including WBC count of >15,000/μl (hazard ratio [HR] = 2.13, *P* = 0.04) and age (HR = 1.03, *P* = 0.008) to be associated with 90-day all-cause mortality. After adjustment for age and WBC count, patients in the highest quartile for TNF-α were 8.3 times more likely to die (*P* = 0.005), and patients in the highest quartile for IL-8 were 4.4 times more likely to die (*P* = 0.01) (Table 3). In contrast, patients in the highest quartile for CCL-5 were 5.5 times more likely to survive (*P* = 0.001) (Table 3).

**TABLE 3:** TNF-α, IL-8 and CCL-5 as independent predictors of 90-day survival in a Cox regression model^*a*^

Biomarker	Quartile	Hazard ratio (95% CI)^*b*^	*P* value
TNF-α	1st (reference)		
	2nd	3.06 (0.57−16.28)	0.18
	3rd	4.52 (0.93−21.87)	0.06
	4th	8.35 (1.86−37.5)	0.005

IL-8	1st (reference)		
	2nd	1.29 (0.30−5.43)	0.72
	3rd	1.55 (0.41−5.80)	0.51
	4th	4.45 (1.38−14.34)	0.01

CCL-5	1st (reference)		
	2nd	0.52 (0.21−1.28)	0.15
	3rd	0.18 (0.05−0.64)	0.008
	4th	0.18 (0.06−0.52)	0.001

a CDI patients were divided into quartiles based on the plasma levels of biomarkers. A Cox proportional hazard model was used to adjust for clinical variables, including age and WBC count at the time of diagnosis.

b The hazard ratio represents the factor by which the hazard changes for each one-unit increase of the cytokine expression. 95% CI, 95% confidence interval, or the upper and lower limits of the confidence interval with a significance level of 0.05.

### Prediction performance of biomarker-based survival and recurrence models.

**FIG 2:**
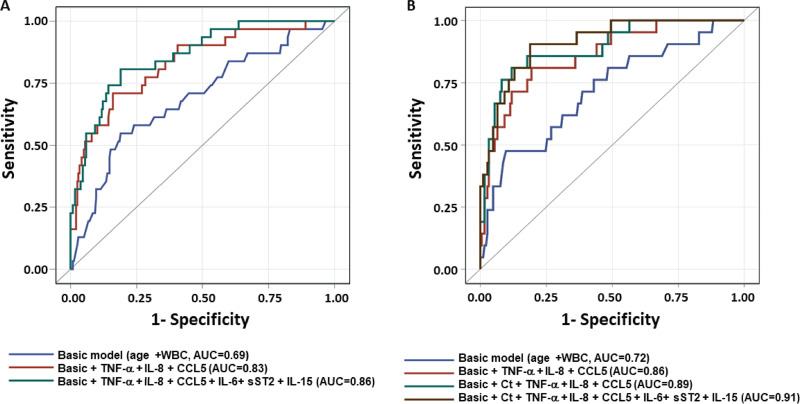
ROC models to predict 3-month survival. Receiver operating characteristic curve analysis was performed using clinical variables and biomarkers most associated with 3-month survival. The basic model comprised of age +plus WBC count. (A) ROC curves of biomarkers associated with survival (*n* = 265). The final model consisted of the basic model + TNF-α + IL-8  +  CCL5  +  IL-6 + sST2  + IL-15. (B) ROC curves of biomarkers in combination with PCR cycle threshold (Ct) data (*n* = 207). The final model consisted of the basic model + Ct + TNF-α + IL-8  +  CCL5 + IL-6 + sST2  + IL-15.

We employed receiver operating characteristic (ROC) curve analysis to assess prediction performance of biomarkers for the mortality and recurrence at 3 months. A basic model without biomarkers comprising age and WBC count showed modest ability to predict 90-day survival (area under the receiver operating characteristic curve [AUC] = 0.69) (Fig. 2A). Inclusion of three independent predictors of survival TNF-α, IL-8 and CCL-5 significantly improved predictive capacity over the basic model (AUC increased to 0.83). Inclusion of all six biomarkers identified through univariate and multivariable analysis (TNF-α, IL-8, CCL-5, IL-6, IL-15 and sST-2) further improved the AUC value to 0.86 (Fig. 2A). However, such improvement was not statistically significant. Further, inclusion of immunosuppression as an additional predictor did not have any influence on the AUC value. PCR cycle threshold (Ct) value was not an independent predictor of survival in the present cohort but improved performance of the model when included (AUC = 0.91) (Fig. 2B). Additionally, although none of these biomarkers independently predicted 90-day recurrence, collectively, they gave an AUC value of 0.77 for 90-day recurrence upon integration with the basic model (see Fig. S1 in the supplemental material).

10.1128/mBio.00905-20.1 FIG S1 ROC models to predict 3-month recurrence. Receiver operating characteristic curve analysis was performed (*n* = 245). The basic model comprises of age + gender + WBC count. The final model consists of the basic model + Charlson index + TNF-α + IL-8 + CCL5 + IL-6 + sST2 + IL-15. Download FIG S1, PDF file, 0.1 MB. Copyright © 2020 Abhyankar et al. 2020 Abhyankar et al. This content is distributed under the terms of the Creative Commons Attribution 4.0 International license.

## DISCUSSION

We investigated association of systemic biomarkers and readily available clinical parameters with survival during CDI. The most important finding from this work was identification of an immune signature that, in combination with basic clinical variables, substantially improved survival prediction. Unadjusted analysis identified several biomarkers (HGF, MIF, IL-4, IL-1β, IL-6, IL-15, and IL-16) that were elevated in severe CDI. Unadjusted analysis of biomarkers between dead and surviving patients identified IL-15, sST-2, IL-8, TNF-α, and IL-6 to be associated with 90-day mortality, whereas CCL-5, EGF, and CCL-4 to be associated with survival. Kaplan-Meier survival analysis showed an association of increased TNF-α, IL-8, IL-6, IL-15, and sST-2 and decreased CCL-5 with all-cause mortality. Cox regression further confirmed these associations for TNF-α, IL-8, and CCL-5. Finally, the biomarker-based model showed excellent predictability for 90-day survival.

Severe CDI was present in 34% patients based on a WBC count of >15,000/μl. A diverse set of circulating biomarkers showed upregulation during severe CDI. These biomarkers included HGF, which is primarily secreted by fibroblasts and plays a major role in wound healing and tissue regeneration (30). Increased levels of IL-4 might also be due to its anti-inflammatory effects. IL-1β was also increased in patients with severe CDI. We had previously reported an increase in IL-1β levels for CDI patients compared to outpatients (16). In a recent study, IL-1β was seen upregulated by toxins A and B *in vitro*, and it was elevated in CDI patients compared to healthy controls (24). Increased serum levels of HGF and IL-1β have also been previously reported in CDI patients compared to outpatients (26). Perhaps most importantly, we have shown in the mouse model of CDI that the IL-1β/Th17 axis plays a major role in promoting inflammatory damage (23).

MIF, IL-15, and IL-16 are pleiotropic cytokines and were seen elevated in severe CDI in this study. These cytokines predominantly support inflammatory immune response. MIF promotes secretion of inflammatory mediators leading to severe pathology (31). Recently, it was shown that MIF levels were increased after CDI both in humans and mice and that neutralization of MIF could protect mice (32). It was suggested that MIF induced a type 17 response to exaggerate CDI, and our results are consistent with this hypothesis. IL-15 is mainly produced by macrophages as well as nonlymphoid cells (33). Monocytes, macrophages, and dendritic cells are the primary targets which in response to IL-15 secrete proinflammatory cytokines, including IL-6, IL-8, and TNF-α (32). IL-15 has been reported to be upregulated in the inflamed tissue from patients with inflammatory bowel disease (IBD) and celiac disease (34). Studies from other laboratories have shown higher levels of IL-15 in CDI patients than in outpatient controls (26) and in patients with severe CDI (24). IL-16 is produced by lymphocytes as well as some epithelial cells and is a chemoattractant for CD4-expressing cells, including T cells, monocytes, dendritic cells, and eosinophils (35). IL-16 was elevated in CDI patients than in healthy donors (24) and was also elevated in patients with Crohn’s disease and ulcerative colitis (36). Thus, MIF, IL-15, and IL-16 may contribute to an exaggerated type 17 response.

In line with our previous studies showing association of IL-6 with mortality (23), higher IL-6 levels correlated with disease severity in this study. Additionally, when patients were divided into quartiles based on IL-6 levels, patients with higher IL-6 (second, third, and fourth quartiles) showed significantly reduced survival probability. Previous reports have shown that serum IL-6 levels were 40-fold higher in CDI patients than in healthy controls (24), and IL-6 was also seen to be associated with severity, although the number of patients analyzed was modest (*n* = 8) (26). We hypothesize that IL-6 might worsen prognosis of CDI because of its ability to promote Th17 cell differentiation.

Overall, there was a substantial increase in proinflammatory cytokines during severe CDI, and many of these, including MIF, IL-1β, and IL-6 were type 17 promoting cytokines. We and others have previously shown that the Th17 axis plays a central role during CDI pathogenesis. The present study confirms these observations and reiterates the Th17 axis as a target for intervention.

In addition to IL-6, five other biomarkers, sST-2, IL-8, IL-15, TNF-α, and CCL-5, showed association with mortality following Kaplan-Meier analysis. Similar to IL-6, higher levels of either sST-2 or TNF-α showed association with an increased mortality. We have previously shown that increased sST-2 levels predicted CDI-associated mortality likely via dysregulation of protective IL-33 signaling (20). In a recent study, systemic TNF-α levels were higher in CDI patients than in healthy donors (24), but TNF-α has not been studied extensively in the context of CDI. In the present study, higher levels of this cytokine (fourth quartile) were indicative of decreased survival by Kaplan-Meier as well as Cox regression analysis. TNF-α is a well-known player in inflammation. C. difficile toxins have been shown to induce TNF-α *in vitro* (37).

IL-8 is a CXC family inflammatory chemokine and the principal human chemoattractant for neutrophils. IL-8 was shown to be secreted by monocytes *in vitro* upon exposure to toxin A (24, 38). A common single nucleotide polymorphism (SNP) in the IL-8 gene promoter was an independent predictor of recurrent CDI (39). IL-8 was also one of the most upregulated biomarkers in CDI patients compared to healthy controls (24), and fecal IL-8 was seen upregulated in severe CDI patients (40). Neutrophils are the primary cells that respond to C. difficile invasion, and neutrophilic inflammation is the hallmark of C. difficile-associated disease. Thus, IL-8 and TNF-α seem to be the robust biomarkers of mortality as identified through univariate and multivariate analyses.

In the present study, EGF, CCL-4, and CCL-5 showed association with protection in univariate analysis. EGF has been reported to exhibit a cytoprotective effect on gastrointestinal epithelia via a receptor-mediated mechanism (41) and also showed inverse association with CDI (26). Similar to EGF, CCL-4 (macrophage inflammatory protein 1β [MIP1β]) showed an inverse correlation with CDI severity and is a chemoattractant for NK cells, monocytes, and other immune cells (26).

CCL-5 (also known as RANTES [42, 43]) was the only biomarker associated with protection in adjusted analysis. Higher levels (second, third, and fourth quartiles) were prosurvival according to Kaplan-Meier analysis. Similarly, patients with higher levels (third and fourth quartiles) were seen protected according to Cox regression analysis. Rao et al. identified CCL-5 as the only biomarker that differentiated between CDI cases and inpatient diarrheal controls and suggested that it was an important mediator of acute intestinal inflammation (26). CCL-5 is expressed by many immune as well as nonimmune cells and actively recruits leukocytes to the inflammatory sites (36, 37). The mechanism of CCL-5-mediated disease modulation remains unknown, and this chemokine warrants further study.

Kaplan-Meier survival analysis did not show association of absolute eosinophil counts (*n* = 338), body mass index (BMI) (*n* = 338), or inflammatory bowel disease status (*n* = 19) with 90-day survival (data not shown). However, patients in the top quartile for absolute neutrophil counts showed decreased probability of survival (*P* = 0.002; data not shown). Eosinophil count at admission was shown to be an independent predictor of mortality (44), and this association was not seen in the present study. Compared to the Kulaylat et al. study (44) which included two centers (*n* = 2,065), the present single-center study had a smaller sample size and looked at survival for the first 90 days after diagnosis, and not in-hospital mortality.

A basic prediction model comprised of age and white blood cell count showed poor prediction ability for survival (AUC = 0.69). Integration of all six biomarkers with the basic model substantially improved the survival prediction capacity of the model (AUC = 0.86). The Charlson comorbidity index did not have any significant impact on survival prediction (data not shown). Inclusion of toxin B PCR Ct values in the survival model comprising biomarkers and clinical parameters further improved the prediction potential (AUC = 0.91).

Currently, there is no biomarker to predict CDI recurrence. Interestingly, when data from all the patients irrespective of their prior CDI history were included in the analysis, CCL-5 was the sole biomarker that showed a strong trend (*P* = 0.07) toward association with 90-day recurrence.

Models based solely on detection of stool toxins A/B (10) or microbiome composition (45) were not able to predict severe CDI or recurrence. Peripheral eosinopenia (44) was identified as an independent predictor of mortality, as was infection with ribotype 027 strain (46). Therefore, an advance from this work is the demonstration that immune profiling adds value to the prediction of mortality in patients with CDI.

This study has several limitations that should be addressed, most importantly that it is retrospective and single site and requires prospective validation before implementation. The study was limited by a lack of information regarding recent history of antibiotic usage, serum albumin levels, toxin status of the infecting strain, microbiome composition, and other chronic inflammatory or major fatal health conditions that could affect systemic cytokine levels. We believe that this information would all be important to include in a prospective evaluation of our model. The study also has significant strengths, most notably being the first to our knowledge to use immune biomarkers to risk stratify patients with CDI for death.

Cytokine-targeted therapies have transformed the treatment of chronic inflammatory diseases, including IBD, providing control of symptoms and longer relapse-free periods (47). We have shown protection from death in murine models by depletion of Th17 cells and by administration of IL-33 (20). The development of predictive models of CDI mortality is an important and necessary first step toward immune therapy of CDI.

## MATERIALS AND METHODS

### Patient population and clinical samples.

CDI patients were retrospectively identified from the University of Virginia (UVA) Medical Center’s electronic database between 2013 to 2016. UVA Medical Center at the time used the Xpert C. difficile PCR (Cepheid, CA, USA) alone for CDI diagnosis. Clinical data were matched to discarded plasma and stool samples that were banked within 48 h of C. difficile testing by the microbiology laboratory. This study evaluated patients with newly diagnosed CDI; patients with a history of CDI within 90 days were excluded. Demographics (age, gender, and race) and clinical characteristics, such as white blood cell count and Charlson comorbidity index, were extracted from the electronic health record database. An immunosuppressant pharmacy grouper was used to identify receipt of immunosuppressants or systemic corticosteroids. Archived and available PCR cycle threshold (Ct) values were collected from the Xpert PCR machine. After initial storage at 4°C for a maximum of 24 h, samples were stored at −80°C until testing. The collection of patient data was approved by the institutional review board (protocol IRB-HSR 16926). This project met the criteria of research involving coded private information or biological specimens.

### Measurement of cytokines in plasma samples.

The concentrations of various target analytes in patient plasma samples were measured on a Bio-Plex 200 suspension array system (Bio-Rad, Hercules, CA, USA) using R&D Systems custom Luminex assays (R & D Systems, Minneapolis, MN). Some analytes, including sST-2 (catalog no. DST 200; R&D Systems), IL-23 (catalog no. D2300B; R&D Systems), and IL-17A (catalog no. HS170; R&D Systems), were measured by using an enzyme-linked immunosorbent assay (ELISA). The analytes that did not give satisfactory signal included IFN-γ, IL-22, IL-13, IL-5, IL-2, and IL-33. All analytes are expressed as picograms per milliliter.

### Outcomes.

The outcomes of interest in this study were 90-day survival and recurrence. Patients were analyzed from the initial CDI diagnosis to death for the outcomes of interest for 90 days or the last time of follow-up, whichever occurred earlier. Due to the small number of events, no further analyses were performed for these outcomes at 30 days.

### Statistical analysis.

Our primary objective was to evaluate effects and predictability of elevated biomarkers on mortality and CDI recurrence. Cytokines in moderate versus severe CDI patient groups were assessed with the Mann-Whitney U test. In survival analysis, patients were categorized into quartiles based on the plasma cytokine levels due to skewed distributions of these biomarkers. Univariate biomarker effect was analyzed using the Kaplan-Meier method, where survival probabilities were estimated and survival differences in the quartiles of each biomarker were evaluated by the log rank test. Those biomarkers with a log rank *P* value of <0.1 were further analyzed using Cox regression, and the final model was determined with stepwise selection for joint effects of these biomarkers on the outcomes. Cox regression analyses were adjusted for demographics and clinical characteristics.

The predictability of biomarkers for the outcomes were estimated using the area under the receiver operating characteristic (ROC) curve. A receiver operating characteristic curve plots the true positive rate (sensitivity) against the false-positive rate (1 – specificity) for all possible cutoff values to predict a dichotomous outcome. In this study, ROC curves were constructed at possible values of demographics, comorbidities, and biomarkers to predict the 90-day mortality and recurrence using logistic regression, and the areas under the ROC curves (AUC) were used to measure predictabilities. Further immunosuppression was also included in evaluating the predictability. A perfect model would have an AUC of 1. The higher the AUC, the better the model is at distinguishing between patients who had the event and those who did not at 90 days. Usually, the optimal cutoff points would be the values of predictors that correspond to the highest sensitivity and lowest specificity. Differences in ROC curves under different models were tested using the empirical method available in SAS (Proc Logistic). Values of *P* < 0.05 were considered statistically significant. The descriptive statistics were analyzed using GraphPad Prism, and survival analyses were performed using SAS 9.4 (SAS Institute Inc., Cary, NC).
