# Supplementary material for: Immune Profiling To Predict Outcome of Clostridioides difficile Infection
Source: mBio. 2020 May 26;11(3):e00905-20. doi: 10.1128/mBio.00905-20 (PMC7251209; doi:10.1128/mBio.00905-20)
Supplement: TABLE S1 [file mBio.00905-20-st001.pdf]

Table S1. Descriptive statistics of biomarkers by mortality at 90 days.

	Alive at 90 days		Dead at 90 days		p-value
	**N**		**N**		
HGF	308	346.64 (166.09, 689.09)	37	329.93 (231.91, 674.16)	0.4864
MIF	308	14068.00 (5845.80, 32148.00)	37	19853.00 (7651.30, 39769.00)	0.3166
IL6	295	8.25 (3.33, 26.48)	36	15.57 (6.38, 25.98)	***0.0404***
IL1b	292	1.56 (1.07, 4.01)	36	1.80 (1.07, 4.53)	0.6079
IL16	308	746.21 (430.60, 1195.90)	37	757.50 (452.87, 1186.50)	0.8219
IL4	308	21.05 (14.60, 61.05)	37	21.05 (14.60, 31.32)	0.1673
IL15	295	2.38 (1.45, 3.70)	36	3.51 (2.02, 5.06)	***0.0115***
EGF	308	191.73 (102.45, 312.96)	37	140.57 (75.87, 225.50)	***0.0278***
sST2	289	174295 (53085, 511656)	35	376634 (185916, 1000000)	***0.0011***
IL23	255	4.88 (4.88, 18.43)	27	4.88 (4.88, 4.88)	0.1736
CCL4	266	2207.90 (1697.10, 2716.50)	33	1808.40 (1300.50, 2324.10)	***0.0067***
IL8	266	71.85 (43.61, 112.71)	33	120.06 (63.78, 173.81)	***0.0131***
TNFa	295	6.78 (4.34, 11.33)	36	9.90 (6.98, 17.54)	***0.0024***
IL17A	255	0.32 (0.09, 0.95)	27	0.50 (0.12, 1.26)	0.2629
IL10	308	4.80 (2.91, 6.25)	37	4.80 (3.33, 7.16)	0.4727
Eotaxin	308	501.91 (310.93, 764.67)	37	572.88 (360.50, 831.56)	0.5231
CCL5	266	35377 (22161, 54040)	33	20650 (10713, 40898)	***0.0025***
