# Supplementary figures and images for: Immune Profiling To Predict Outcome of Clostridioides difficile Infection
Source: mBio. 2020 May 26;11(3):e00905-20. doi: 10.1128/mBio.00905-20 (PMC7251209; doi:10.1128/mBio.00905-20)

**Figure- S1. ROC models to predict 3- month recurrence.**

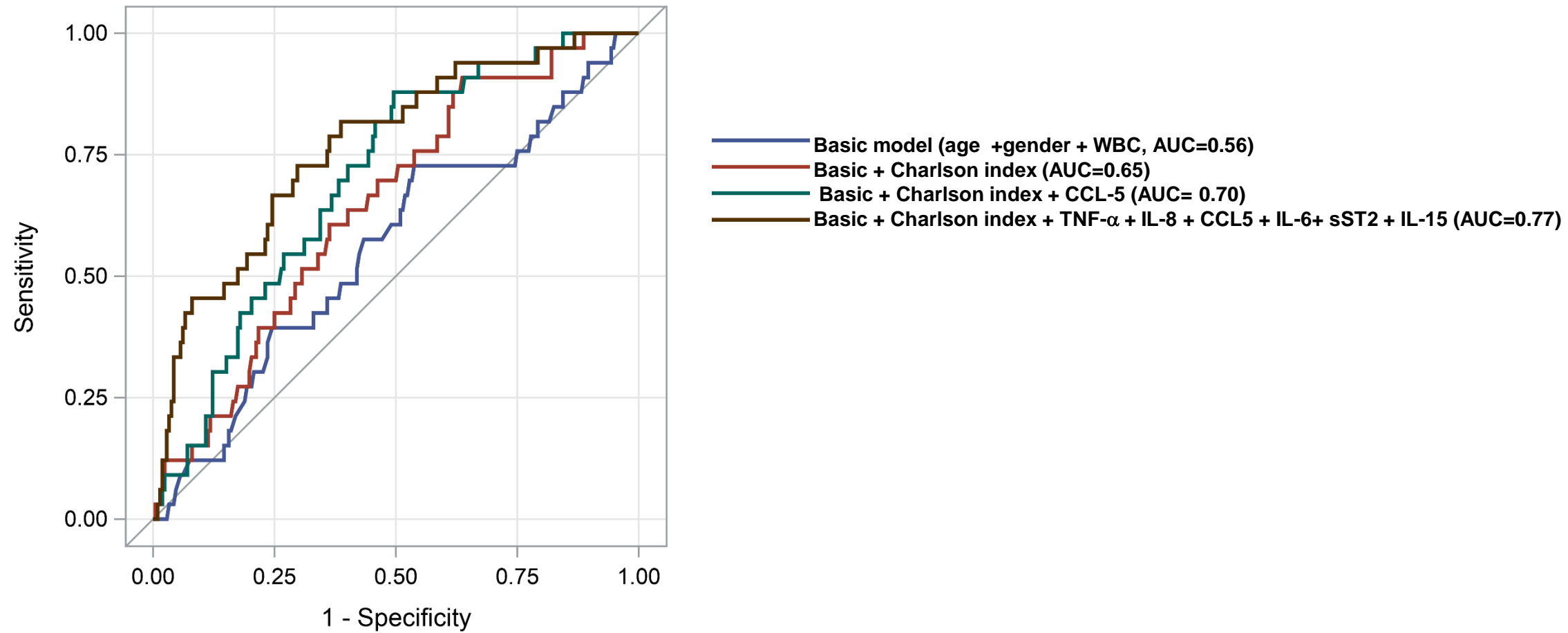

Supplement: FIG S1 [file mBio.00905-20-sf001.pdf]
